# Intubation during a medevac flight: safety and effect on total prehospital time in the helicopter emergency medical service system

**DOI:** 10.1186/s13049-020-00784-z

**Published:** 2020-09-07

**Authors:** Hiroki Maeyama, Hiromichi Naito, Francis X. Guyette, Takashi Yorifuji, Yuki Banshotani, Daisaku Matsui, Tetsuya Yumoto, Atsunori Nakao, Makoto Kobayashi

**Affiliations:** 1 Department of Emergency, Critical Care, and Disaster Medicine, Okayama University Graduate School of Medicine, Dentistry and Pharmaceutical Sciences, 2-5-1 Shikata, Okayama, 700-8558 Japan; 2 Department of Emergency and Critical Care Medicine, Tsuyama Chuo Hospital, Tsuyama, Japan; 3 Department of Emergency Medicine, University of Pittsburgh School of Medicine, Pittsburgh, PA USA; 4 Department of Epidemiology, Okayama University Graduate School of Medicine Dentistry and Pharmaceutical Sciences, Okayama, Japan; 5 Tajima Emergency and Critical Care Medical Center, Toyooka Public Hospital, Toyooka, Japan

**Keywords:** Transportation, Airway management, Air ambulance, Time-to-treatment

## Abstract

**Introduction:**

The Helicopter Emergency Medical Service (HEMS) commonly intubates patients who require advanced airway support prior to takeoff. In-flight intubation (IFI) is avoided because it is considered difficult due to limited space, difficulty communicating, and vibration in flight. However, IFI may shorten the total prehospital time. We tested whether IFI can be performed safely by the HEMS.

**Methods:**

We conducted a retrospective cohort study in adult patients transported from 2010 to 2017 who received prehospital, non-emergent intubation from a single HEMS. We divided the cohort in two groups, patients intubated during flight (flight group, FG) and patients intubated before takeoff (ground group, GG). The primary outcome was the proportion of successful intubations. Secondary outcomes included total prehospital time and the incidence of complications.

**Results:**

We analyzed 376 patients transported during the study period, 192 patients in the FG and 184 patients in the GG. The intubation success rate did not differ between the two groups (FG 189/192 [98.4%] vs. GG 179/184 [97.3%], *p* = 0.50). There were also no differences in hypoxia (FG 4/117 [3.4%] vs. GG 4/95 [4.2%], *p* = 1.00) or hypotension (FG 6/117 [5.1%] vs. GG 5/95 [5.3%], *p* = 1.00) between the two groups. Scene time and total prehospital time were shorter in the FG (scene time 7 min vs. 14 min, *p* <  0.001; total prehospital time 33.5 min vs. 40.0 min, *p* <  0.001).

**Conclusions:**

IFI was safely performed with high success rates, similar to intubation on the ground, without increasing the risk of hypoxia or hypotension. IFI by experienced providers shortened transportation time, which may improve patient outcomes.

## Background

The Helicopter Emergency Medical Service (HEMS) typically intubates patients who need advanced airway management prior to takeoff. In-flight intubation (IFI) is often avoided because it is considered difficult due to limited space, difficulty communicating, and vibrations and gravitational forces in flight [1]. Endotracheal intubation (ETI) is a multistep process that includes pre-oxygenation, endotracheal tube preparation, establishing intravenous access, and administration of induction medications [2]. Moreover, it takes more time to complete ETI and secure both the endotracheal tube and the patient during emergency care. Intubation prior to takeoff may increase the total prehospital time. IFI may shorten the total prehospital time, as the procedure is conducted during the flight, and in some patients, this time difference may alter outcomes [3].

IFI has been described in only a few reports. Harrison and colleagues found no difference in HEMS IFI success rates as compared with ETI in the field and in-hospital ETI. Paramedic flight crews completed ETI with a success rate of 96.4% [4]. Thomas and colleagues analyzed flight crew airway management in four different settings (in flight, at the trauma scene, in ambulances, and at hospitals) and found that airway management success rates were comparable, even in the in-flight setting [5]. However, the rate of complications associated with IFI has not been reported. Moreover, the prehospital time typical for IFI has not been described. We tested whether IFI can be successfully performed without increases in complications and whether IFI shortens total prehospital time as compared with intubation on the ground.

## Methods

We performed a single-center, retrospective, cohort study. Patients were treated from April 1, 2010 to March 31, 2017 by the Toyooka Hospital HEMS, and data were obtained from hospital medical charts. The Toyooka Hospital Ethical Committee approved the study. The requirement for informed consent was waived. Study results are presented according to the STROBE guidelines for observational studies.

### HEMS system

**Fig. 1:**
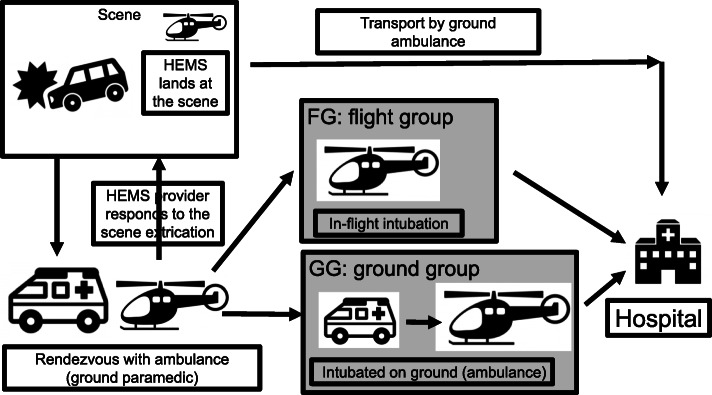
Patient flow chart and grouping. Patients in the study were separated into 2 groups. Flight group (FG) patients were intubated during the flight; ground group (GG) patients were intubated on the ground prior to takeoff. The HEMS system receives a dispatch request from a public EMS, then takes off from the hospital and lands at the rendezvous point (RP). In some cases, the HEMS lands at the scene. Generally, at the RP, the HEMS staff make contact with the patient transported by the ground ambulance, treat the patient, then transport the patient to the hospital. If the HEMS team reaches the RP before ground EMS, the HEMS staff may respond to the scene. EMS: emergency medical service, HEMS: helicopter emergency medical service

In the Japanese HEMS system, each base tertiary medical center employs one helicopter. Typically, the HEMS system receives a dispatch request from a ground Emergency Medical Service (EMS); then, a HEMS helicopter takes off from the hospital and lands at a predefined rendezvous point (RP). At the RP, the HEMS staff contacts the ground ambulance crew, stabilizes the patient, then transports the patient to the hospital. If the HEMS team reaches the RP before ground EMS, the HEMS staff may move from the RP to the scene to reach the patient (Fig. 1).

### Service area and protocol of Toyooka HEMS system

**Fig. 2:**
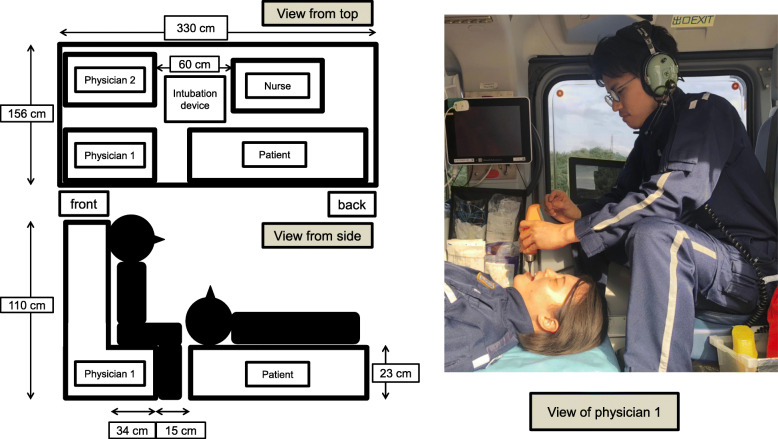
Map of the cabin and positioning of providers performing in-flight intubation. Cabin and provider seat sizes are shown. Physician 1 oxygenates the patient, secures the airway, and intubates the patient. Devices for IFI are set on the left side of Physician 1. Nurse and Physician 2 prepare and administer medications. They also support the procedure. The use of video laryngoscopy or direct laryngoscopy for intubation is at the discretion of the attending physician. Physicians preferred to use the video laryngoscope for IFI presumably due to the limited space of the cabin

The Toyooka HEMS system is responsible for the northern region of Hyogo Prefecture, the northern region of Kyoto Prefecture, and the eastern region of Tottori Prefecture, covering an area of approximately 6226 km^2^ with a population of approximately 784,000 people. This area is rural and mountainous with only a few hospitals. The EC135 helicopter used by the Toyooka HEMS accommodates a maximum of seven passengers. Commonly, six passengers—a pilot, a mechanic, two physicians, a nurse, and the patient—are on board (Fig. 2). The HEMS physicians are thoroughly trained in airway management and ETI in the emergency department, intensive care unit, and operating room prior to assignment to the HEMS. Their training also includes ETI using a simulator for both video and direct laryngoscopy on the helicopter.

Most severe cases are treated by the HEMS system including patients with stroke, cardiovascular disease, sepsis, trauma, or cardiac arrest. ETI is performed for airway obstruction, regurgitation, respiratory failure (< percutaneous oxygen saturation [SpO_2_] 90%), circulatory failure (< systolic blood pressure [sBP] 90 mmHg), and coma (Glasgow Coma Scale score < 8). Patients are intubated using sedatives (midazolam or ketamine), analgesics (fentanyl), and neuromuscular blockade (rocuronium or vecuronium). Rapid sequence induction was applied in most patients. The Pentax Airway Scope® (AWS-S100®; Pentax Corporation, Tokyo, Japan) was used for video laryngoscopy. The attending physician could freely choose to use either direct laryngoscopy or video laryngoscopy.

### Patient selection and data collection

**Fig. 3:**
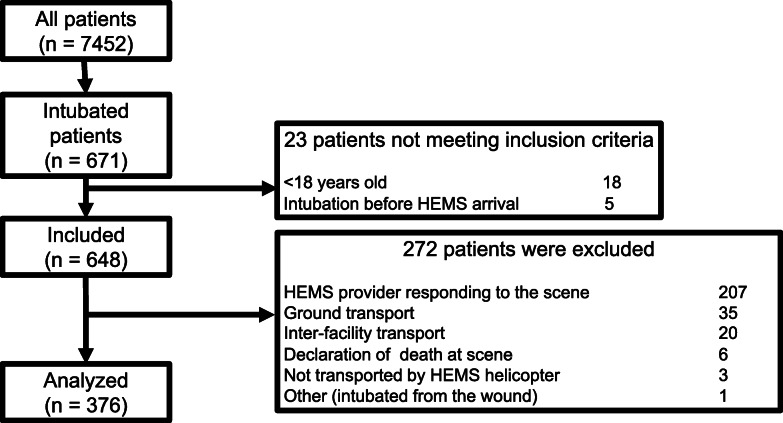
STROBE diagram detailing the inclusion and exclusion criteria

Patients over 18 years of age who were intubated by a Toyooka HEMS physician in the prehospital setting were included in this analysis. Exclusion criteria were interfacility transport, ground transport, declaration of death at the scene, and not transported by HEMS helicopter. We also excluded patients in whom total prehospital time was confounded by long extrication times or other procedures (Fig. 3).

Successful ETI attempts were verified by auscultation and end tidal carbon dioxide measurement. We divided patients into two groups for analysis. In the flight group (FG), patients were intubated after takeoff during the flight. In the ground group (GG), patients were intubated on the ground prior to takeoff, usually in the ambulance at the RP (Fig. 1).

The following measured data was collected: age, sex, etiology (medical conditions / “trauma and others”). Medical conditions included heart disease, respiratory disease, stroke, and sepsis. The classification “trauma and others” included trauma and suffocation. For the ETI procedure, we recorded success rate, number of attempts, use of video laryngoscopy or direct laryngoscopy, training of the emergency physician who performed the intubation, and whether the patient experienced hypoxia or hypotension during intubation. We recorded scene time (time from HEMS staff arrival at the RP to patient loading onto the helicopter) and total prehospital time (time from when the helicopter left the base hospital to when it arrived with the patient at the destination hospital). We defined hypoxia as the patient’s SpO_2_ dropping below 90% and hypotension as sBP below 90 mmHg during intubation [6–9]. The primary outcome was the proportion of successful ETI. Secondary outcomes included scene time, total prehospital time, and incidence of hypoxia or hypotension.

### Data analysis

Continuous variables were described as medians with interquartile range and compared using the Mann–Whitney U-test. Categorical variables were described as numbers or percentages and compared using Fisher’s exact test. All statistical analyses were performed with EZR version 1.40 (Saitama Medical Center, Jichi Medical University; http://www.jichi.ac.jp/saitama-sct/SaitamaHP.files/statmed.html; Kanda, 2012), which is a graphical user interface for R (The R Foundation for Statistical Computing, Vienna, Austria, version 2.13.0) that was modified from R commander (version 1.6–3) to add functions frequently used in biostatistics [10]. All *p*-values were two sided, and *p* <  0.05 was considered statistically significant.

## Results

Of the 7452 patients treated by Toyooka HEMS during the study period, 671 were intubated. We excluded 295 patients who met the exclusion criteria (Fig. 3). Finally, 376 patients with a median age of 74 years (IQR 60–82) were analyzed. Of the 376 patients, 192 (51.1%) were intubated in flight (FG) and 184 (48.9%) were intubated prior to takeoff (GG) (Fig. 3). The groups did not differ with respect to age, sex, injury type, and vital signs at the scene (Table 1). However, the more patients in the GG had respiratory disease than in the FG (6% vs. 1.6%, *p* = 0.03).

**Table 1:** Patient characteristics for the two groups

	Flight Group	Ground Group	*p*-value
	(*n* = 192)	(*n* = 184)	
Age, median (IQR), y	74 (60–82)	73 (59–83)	0.91
Male, No. (%)	135 (70.3)	115 (62.5)	0.13
Medical conditions, No. (%)	108 (56.3)	105 (57.1)	0.92
Heart disease	45 (23.4)	40 (21.7)	0.71
Respiratory disease	3 (1.6)	11 (6.0)	0.03
Stroke	35 (18.2)	37 (20.1)	0.70
Sepsis	2 (1.0)	0 (0)	0.50
Others	23 (12.0)	17 (9.2)	0.41
Trauma and others, No. (%)	84 (43.7)	79 (42.9)	0.92
Trauma	55 (28.6)	48 (26.1)	0.64
Suffocation	11 (5.7)	5 (2.7)	0.20
Others	18 (9.4)	26 (14.1)	0.20
Vital sign, median (IQR)^a^			
Respiratory rate	22 (18–30)	22 (16–28)	0.56
Heart rate	100 (80–126)	89 (70–120)	0.08
Systolic blood pressure	130 (80–180)	129 (83–189)	0.80
Glasgow Coma Scale	6 (3–11)	6 (3–7)	0.19
CA, No. (%)	75 (39.1)	89 (48.4)	0.08

*Abbreviations*: *CA* Cardiac arrest, *IQR* Inter quartile range

^a^excluding cardiac arrest

### ETI success rate, complications, and mortality

The overall intubation success rate was not different between two groups (FG 189/192 [98.4%] vs. GG 179/184 [97.3%], *p* = 0.50). First-pass success rate tended to be higher in the GG, but the difference was not significant (FG 88.5% vs. 93.5%, *p* = 0.11) (Table 2). There were five cases of failed intubation requiring cricothyrotomy, 1 in the FG and 4 in the GG, and bag-valve-mask ventilation was performed in 2 patients in the FG and one patient in the GG. There were no differences in the experience of the emergency services physician (FG 4.0 years vs. GG 4.0 years, *p* = 0.38) or in the physicians’ accumulated ETI experience between the groups (Table 2). Video laryngoscopy was used significantly more frequently in the FG than in the GG (FG 160/192 [83.3%] vs. GG 35/184 [19.0%], *p* <  0.001). In patients who were not experiencing cardiac arrest, there were no differences in the incidence of hypoxia or hypertension between the 2 groups (Table 2). Mortality was significantly higher in the GG (109/184, 64.5%) than in the FG (86/192, 45.7%; *p* = 0.01).

**Table 2:** ETI success rate, characteristics, complications, and death

	Flight Group	Ground Group	*p*-value
Successful cases, No. (%)	189/192 (98.4)	179/184 (97.3)	0.50
Number of ETI attempts, No. (%)			
First pass	170/192 (88.5)	172/184 (93.5)	0.11
Second pass	189/192 (98.4)	178/184 (96.7)	0.33
Third pass		179/184 (97.3)	
Video laryngoscopy, No. (%)	160/192 (83.3)	35/184 (19.0)	< 0.001
Cricothyroidotomy, No. (%)	1/192 (0.5)	4/184 (2.2)	0.21
Physician years specializing in emergency services, median (IQR), years	4 (3–6)	4 (3–5)	0.38
Physician accumulated experience with intubations, No. (%)			
> 1000	79 (41)	82 (45)	0.53
501–1000	35 (18)	36 (20)	0.79
101–500	78 (41)	66 (36)	0.40
Complications, excluding CA cases, No. (%)			
Hypoxia	4/117 (3.4)	4/95 (4.2)	1.00
Hypotension	6/117 (5.1)	5/95 (5.3)	1.00
Deaths, No. (%)	86/192 (45.7)	109/184 (64.5)	0.01

*ETI* Endotracheal intubation, *CA* Cardiac arrest, *IQR* Interquartile range

### Time elapsed prior to hospital arrival

Finally, we examined whether IFI would affect how quickly the patient could be treated at the hospital. There was no difference in time from hospital takeoff to RP arrival between the 2 groups (FG 13 min vs. GG 13 min, *p* = 0.43). Scene time was approximately 7 min shorter in the FG (FG 7 min vs. GG 14 min, *p* <  0.001). Total prehospital time was also shorter for the FG (FG 33.5 min vs. GG 40.0 min, *p* <  0.001) (Table 3).

**Table 3:** Prehospital time

	Flight Group	Ground Group	*p*-value
	(*n* = 192)	(*n* = 184)	
Takeoff to RP arrival, median (IQR), min	13 (10–16)	13 (9–17)	0.43
Prehospital activity time, median (IQR), min	7 (5–9)	14 (11–17)	< 0.001
Total prehospital time, median (IQR), min	33.5 (28–40)	40 (33–47)	< 0.001

*Abbreviations*: *RP* Rendezvous point, *IQR* Interquartile range

## Discussion

This is the first report to describe the safety of IFI in the context of associated complications (hypoxia and hypotension) and the impact of IFI on total prehospital time. The success rate for intubation in the helicopter was high (98.4%) and was similar to the success rates of prehospital ETI by physicians reported in other studies. (Table 4) [7, 11–14]. Additionally the overall ETI success rate did not differ between the FG and GG. Moreover, the incidences of hypoxia and hypotension were similar between the groups and consistent with previously published complication rates [7, 11, 13, 15]. Our data indicate that ETI can be safely conducted by experienced providers during flight.

**Table 4:** Comparative data of prehospital intubation by physician from the present and previous studies

	Helm M, et al. [7] (2013)	Sunde GA, et al. [11] (2015)	Piegeler T, et al. [12] (2016)	Caruana E, et al. [13] (2015)	Kamiutsuri K, et al. [14] (2013)	This study (2020)
Number	150	2144	988	1251	742	376
Age, y	40 (21–61)^a^	53 (0–95)^a^	49.7 (25.7–65.9)^a b^	60.3 (18.6)^d^	CA 61.8 (20.9)^d^	74 (60–82)^e^
			52.7 (34.5–66.5) ^a c^		Non CA 50.7 (20.4)^d^	
Medical (%)	22.0	55.0	NA	NA	68% (CA), 13.6% (Non-CA)	56.4
Trauma (%)	78.0	44.0	NA	NA	32% (CA), 86.4% (Non-CA)	27.7
CA (%)	0.0	42.0	46.4	57.4	61.3	44.0
Success rate (%)						
Total	100.0	98.7	99.5	99.5	99.1	97.9
First pass	92.0	85.5	96.4	63.8	NA	91.8
Second or additional passes	8.0	13.2	3.1	35.7	NA	7.7
Hypoxia, excluding CA (%)	12.0	2.1	NA	10.0	NA	3.7
Hypotension, excluding CA (%)	NA	3.0	NA	1.3	NA	5.2

*Abbreviations*: *CA* Cardiac arrest, *IQR* Interquartile range. ^a^ median (range) ^b^ first attempt success ^c^ two or more attempts success ^d^ mean (SD) ^e^ median (IQR)

Mortality is reduced almost 50% by treating patients in the field as compared with patients treated later in a trauma center [16]. However, prehospital ETI may prolong prehospital time by 12 min, leading to a delay in definitive care [17]. Lansom and colleagues reported that prehospital intubation prolonged total prehospital time by 25 min, but shortened the time from arrival at the emergency department to the initiation of computed tomography imaging by 11 min [18]. In patients with heart disease, mortality was decreased to an adjusted odds ratio of 0.49–0.73 when total prehospital time was shortened [19]. Trauma mortality is decreased by shortening the scene time to < 50% of the total prehospital time [20]. In our study, intubating patients in the helicopter decreased scene time by 7 min. Similarly, Nakstad and colleagues reported that the scene time was prolonged by approximately 8 min when ETI was performed [21]. Additionally, when patients arrive intubated to the emergency department, it may facilitate critical examinations or interventions. Further research is needed to determine if the 7-min shortening that we observed decreases mortality.

In the current study, approximately half of patients were intubated using video laryngoscopy, with a significantly larger proportion in the FG. Past studies describing IFI were conducted in the 1990s before the widespread adoption of the video laryngoscope [4, 5], and therefore, did not address the use of the device. During flight, the distance between the physician performing ETI and the head of patient is approximately 15 cm. The fixed low position of the stretcher and the position of the physician’s knee near the patient’s head may make ETI using direct laryngoscopy difficult. Video laryngoscopy may have provided the HEMS physicians with an improved laryngoscopic view and contributed to the observed high success rates. Additionally, in the helicopter, video laryngoscopy allows providers to share their view and may have improved communication with the assisting providers, which is typically limited to communication through headsets given the noisy environment. Finally, the ergonomics and thicker blade of the video laryngoscope may have improved the intubation conditions, given the vibration in the aircraft. Because it is difficult to confirm ETI by auscultation in flight, the ability of the video laryngoscopy to provide capnography and direct visualization facilitates safe confirmation of airway management after takeoff.

Intubation success generally depends on the experience of the provider [22, 23]. ETI success rates are higher when a physician performs the procedure as compared with paramedics or nurse providers [24–26], which may partly explain the high IFI success rate we observed. However, 2 prior studies of IFI with experienced paramedics performing the procedure had success rates exceeding 95%, indicating the feasibility of IFI by other experienced providers (Table 5). Because the incidence of hypoxia and hypotension in our study did not differ from that seen previously, our findings support that safety will be maintained when intubation is performed by an experienced provider [7, 11, 13]. The providers’ experience and the air medical education programs available should be carefully considered before the introduction of IFI in a HEMS system. Moreover, patient selection should be limited to patients with time-dependent conditions that justify intubation in the aircraft. We believe that adapting IFI to flight crews with different provider compositions will require further study and verification.

**Table 5:** Comparative data of in-flight intubation from the present and previous studies

	Harrison T, et al. [4] (1997)	Thomas SH, et al. [5] (1999)	This study (flight group) (2020)
Number	120	246	192
Age, y	27 (2–75)^a^	NA	74 (60–82)^b^
Medical (%)	23.0	NA	56.3
Trauma (%)	77.0	NA	28.6
CA (%)	42.0	NA	39.1
Success rate (%)			
Total	94.2	95.5	98.4
First attempt	75.0	71.9	88.5
Two or more attempts	19.2	23.6	9.9
Hypoxia, excluding CA (%)	NA	NA	3.4
Hypotension, excluding CA (%)	NA	NA	5.1

*Abbreviations*: *CA* Cardiac arrest, *IQR* Interquartile range. ^a^ median (range) ^b^ median (IQR)

Our investigation has several limitations. The study was performed using data from a single HEMS with ETI performed only by highly trained physicians; therefore, the results may not be generalizable to other EMSs. We could not obtain intubation times for these patients; however, scene time may be a surrogate for estimating procedure time. We did not adjust for patient mortality between the groups due to the diversity of patients and the retrospective nature of the study. Patients with more severe conditions may have been intubated before flight more frequently, which may explain why the proportion of patients with cardiac arrest or respiratory disease was higher in the GG. Finally, we could not examine whether the decreased prehospital time afforded by IFI in this study translated into improved outcomes.

## Conclusions

IFI was safely performed with high success rates, similar to intubation on the ground, without increasing the risk of hypoxia or hypotension. IFI by experienced providers decreased total prehospital time. Further studies are needed to determine if this strategy is associated with improved patient outcomes.

## Data Availability

Data was available upon reasonable request.

## Abbreviations

EMS: Emergency Medical Service
ETI: Endotracheal intubation
FG: Flight group
GG: Ground group
HEMS: Helicopter Emergency Medical Service
IFI: In-flight intubation
sBP: Systolic blood pressure
SpO2: Percutaneous oxygen saturation
RP: Rendezvous point
